# Crisis-driven cholera resurgence switches focus to oral vaccine

**DOI:** 10.2471/BLT.18.020718

**Published:** 2018-07-01

## Abstract

Oral rehydration was once the mainstay of treatment for cholera, but today’s cholera outbreaks fuelled by conflict and instability require a new approach. Sophie Cousins reports.

On a hot, humid afternoon at the world’s largest diarrhoeal disease hospital, dozens of patients are filing in, many being carried in the arms of loved ones, frail and barely alive.

Inside the Cholera Hospital – as it is commonly known – in Dhaka, at the icddr,b (formerly the International Centre for Diarrhoeal Disease Research, Bangladesh or ICDDR, B), hundreds of patients receive rehydration treatment.

Up to 1000 patients can be admitted each day at this time of the year, as the rains peak and temperatures soar. Outside the hospital, the ward has extended into circus-size tents in the car park. Most patients recover quickly and go home within 24 hours.

“We don’t say no to anyone and we don’t charge anyone,” says Dr Azharul Islam Khan, who is the chief physician and head of hospitals at the icddr,b.

The bacterium *Vibrio cholerae* has wreaked havoc for hundreds of years. Originating in the Ganges delta in India, the first recorded cholera epidemic started in 1817 and travelled along trade routes through Asia and to the shores of the Caspian and Mediterranean seas. Since then, regular outbreaks across the world have killed millions of people.

Cholera is an acute diarrhoeal infection caused by ingesting food or water contaminated with *Vibrio cholerae*. If left untreated, the infection can kill within hours. Each year between 1.3 to 4 million cases, and up to 143 000 deaths are reported to the World Health Organization (WHO). But the true burden of cholera is unknown.

“Reporting of cholera is not reliable. The number of cholera cases reported to us is considered to be the tip of the iceberg,” says Dr Dominique Legros, from WHO’s health emergencies programme.

There are many reasons for this, he says. For one, it’s difficult to confirm cases in large outbreaks where diagnostic capacity is limited. Second, the symptoms of less severe cholera are similar to those of other diarrhoeal diseases. Third, some countries may be reluctant to report cases of cholera for fear this will affect trade or tourism, Legros adds.

Bangladesh, an impoverished country of 162 million people, where cholera is endemic, has been at the forefront of the global fight against this ancient disease.

In the past 30 years, oral rehydration solution (ORS) – a mixture of salt, sugar and clean water – has saved an estimated 50 million lives worldwide, particularly those of children who are most vulnerable to diarrhoea-related dehydration.

The simple and inexpensive mixture was first formulated to treat cholera by researchers at the icddr,b in Dhaka and their colleagues at the Johns Hopkins Center for Medical Research and Training in Kolkata, India in the late 1960s.

“ORS is the mainstay in the prevention of dehydration,” Khan says. “Bangladesh has come a long way in terms of promoting ORS and raising awareness about how to treat cholera.”

ORS has helped Bangladesh to make huge strides in improving child health in recent decades. From 1988 to 1993, diarrhoea was the cause of almost one in five deaths among children under the age of five years. Between 2007 and 2011, only 2% of these deaths were related to diarrhoea, according to the Bangladesh* Demographic and health survey 2011*.

Today the United Nations Children’s Fund distributes around 500 million ORS sachets a year in 60 low and middle-income countries at a cost of around US$ 0.10 each.

Yet, while ORS has saved millions of lives, cholera shows no sign of waning, even in the region where it originated. Cholera still persists for very simple reasons: a lack of access to safe water, and poor sanitation and hygiene.

For Munirul Alam, senior scientist at the Infectious Diseases Division at the icddr,b, people living in conditions of overcrowding, poor hygiene and lack of access to safe water risk contracting cholera.

Around the world, as wars, humanitarian crises and natural disasters, such as flooding and droughts, uproot millions of people, destroy basic services and health-care facilities, cholera is surging.

Cholera broke out in conflict-torn Yemen almost two years ago. It has since claimed almost 2500 lives and infected about a million people in the country of 30 million. In Nigeria, three cholera outbreaks have already been declared this year in the country’s north-east, where millions have been displaced by conflict.

If ORS is so effective in preventing death, why are people still dying of cholera? “It’s the problem of access to care,” Legros says. “Cholera starts as acute diarrhoea and can quickly become extremely severe.”

“In emergency situations, where hospitals have been destroyed, are inaccessible or lack the basic resources, people with severe dehydration do not always receive intravenous rehydration treatment that they need.”

Severely dehydrated people need the rapid administration of intravenous fluids plus ORS during treatment, along with appropriate antibiotics to reduce the duration of diarrhoea and reduce the *V. cholerae* in their stool.

In Yemen, Dr Nahla Arishi, paediatric co-ordinator at Alsadaqah Hospital in Aden, a port city in the south of the country, is treating up to 300 cholera cases a day.

Last year the Yemeni paediatrician travelled to Dhaka’s icddr,b to participate in a week-long training on cholera and malnutrition case management and take back the skills and knowledge to her country.

Arishi, one of a team of 20 doctors and nurses from Yemen, learnt about the assessment of dehydration, food preparation, severe acute malnutrition and observed how the Cholera Hospital manages patients.

“They will be acting as good master trainers,” Khan says, adding that the icddr,b regularly deploys its experts to assist WHO and governments with the response to diarrhoeal diseases in emergencies.

While Arishi brought knowledge home with her, there are limits in applying these lessons. Battling cholera in Yemen is extremely challenging and the situation differs from that in Bangladesh.

Alsadaqah Hospital has ORS and intravenous fluids but the provision of these simple services is constantly disrupted – disruptions that can mean a matter of life and death, she says. “Electricity is on and off and is worse in summer, [it’s the] same with water [supplies].”

In emergencies such the one in Yemen, the oral cholera vaccine is playing an increasingly important role. 

Currently there are three WHO pre-qualified oral cholera vaccines, two of which are used in areas experiencing outbreaks. They require two doses at least 14 days apart and can provide protection for up to five years.

In the last five years the use of these vaccines has increased exponentially, Legros says. The reason being that the vaccine is available, easy to use, well tolerated and addresses “a disease which people fear a lot.” “If you come with a vaccine, people will take it,” he says.

In 2013, WHO established a stockpile of two million doses of oral vaccine financed by Gavi, the Vaccine Alliance, to respond to cholera outbreaks and to reduce the risk of outbreaks in humanitarian crises. 

These settings include refugee camps, such as those for the Rohingyas in south-eastern Bangladesh, where two vaccination campaigns were completed between October and November 2017 and in May of this year.

The oral cholera vaccine has also been deployed in outbreaks in Haiti, Iraq and South Sudan, and recently in Malawi and Uganda.

“We’ve just started using the vaccine as a first stop for sustainable cholera control, followed up with water, sanitation and hygiene (WASH) interventions,” Legros says, referring to WASH measures that include improved water supply and sanitation, provision of safe drinking water and handwashing with soap.

But, Firdausi Qadri, a vaccine scientist and acting senior director of icddr,b’s Infectious Diseases Division, warns there aren’t enough vaccines stockpiled.

Last year an ambitious strategy to reduce cholera deaths by 90% by 2030 was launched by the Global Task Force on Cholera Control, a partnership of more than 30 health and development organizations including WHO, established in 2011. 

According to *Ending cholera: a global roadmap to 2030,* which targets 47 countries, prevention and control can be achieved by taking a multisectoral approach and by combining the use of oral cholera vaccines with basic water, sanitation and hygiene services in addition to strengthening health-care systems and surveillance and reporting.

The roadmap also calls for a focus on cholera “hotspots,” places that are most affected by cholera – like the high risk areas in Bangladesh – that play an important role in the spread of cholera to other regions.

Bangladesh now has plans for a more systematic prevention and control of cholera, in line with the global strategy. To boost oral cholera vaccine supplies in the country, a local company is producing a vaccine, via technology transfer from India, and this could result in up to 50 million doses a year for the country, Qadri says.

But she recognizes that greater reliance on the vaccine will come at the expense of investing in water and sanitation hygiene services.

“Water, sanitation and hygiene interventions are what we need,” she says. “We have to change the whole environment and we have to educate people.”

Dr Khairul Islam, country director of WaterAid Bangladesh, agrees.

“No one would disagree that water, sanitation and hygiene interventions are ultimately the best way to prevent cholera and other water-borne gastrointestinal diseases,” he says.

**Figure:**
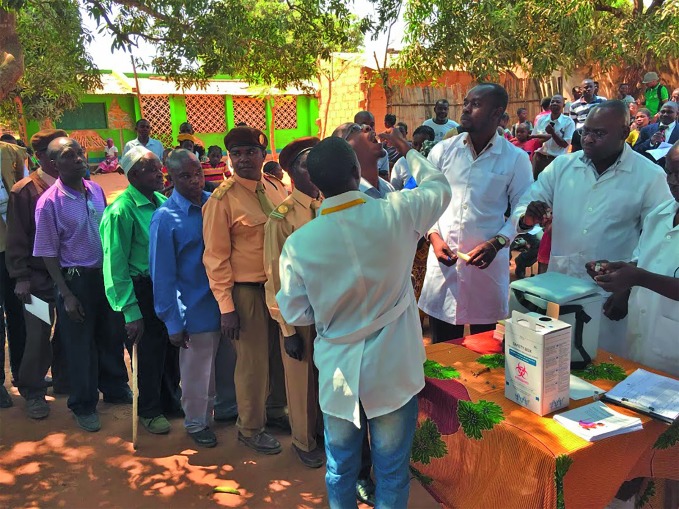
Residents queue up to receive the oral cholera vaccine in the city of Nampula in Mozambique during the vaccination campaign in 2016.

WHO/L. Pezzoli

**Figure:**
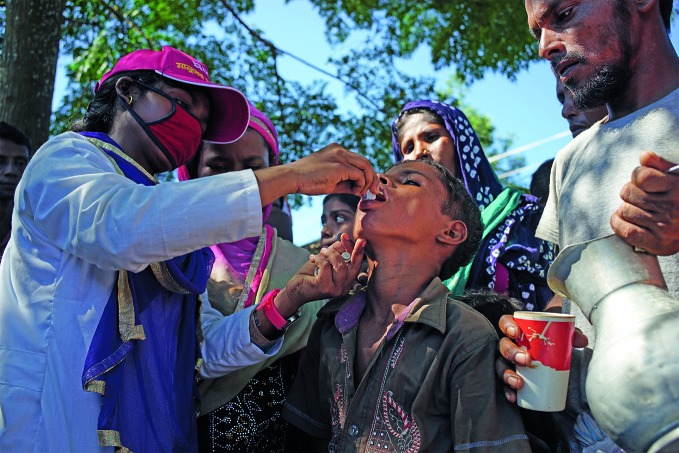
Rohingas in Cox’s Bazaar in south eastern Bangladesh receive the oral cholera vaccine in 2017.

WHO/W. Owens

